# Foodborne Zoonoses Common in Hunted Wild Boars

**DOI:** 10.1007/s10393-020-01509-5

**Published:** 2020-12-16

**Authors:** Maria Fredriksson-Ahomaa, Laura London, Teresa Skrzypczak, Tuija Kantala, Ilona Laamanen, Mia Biström, Leena Maunula, Tuija Gadd

**Affiliations:** 1 Department of Food Hygiene and Environmental Health, Faculty of Veterinary Medicine, University of Helsinki, P.O.Box 66, 00014 Helsinki, Finland; 2 Virology Unit, Finnish Food Authority, Helsinki, Finland; 3 Veterinary Bacteriology and Pathology Unit, Finnish Food Authority, Helsinki, Finland

**Keywords:** Foodborne pathogens, Infectious diseases, Wild boar, Public health, Serology, Hygiene, Hunting

## Abstract

The northern European wild boar population has increased during the last decade. Highest wild boar numbers in Finland have been reported in the southeastern part near the Russian border. Wild boars may be infected with several human and animal pathogens. In this study, we investigated the presence of important foodborne pathogens in wild boars hunted in 2016 in Finland using serology, PCR and culturing. Seroprevalence of *Salmonella* (38%) and *Yersinia* (56%) infections was high in wild boars. Antibodies to hepatitis E virus, *Toxoplasma gondii* and *Brucella* were found in 18%, 9% and 9% of the wild boars, respectively. *Trichinella* antibodies were detected in 1% of the animals. We recorded no differences in the seroprevalence between males and females. However, *Yersinia* and *T. gondii* antibodies were detected significantly more often in adults than in young individuals. *Listeria monocytogenes* (48%) and *stx*-positive *Escherichia coli* (33%) determinants were frequently detected in the visceral organs (spleen and kidneys) by PCR. *Yersinia pseudotuberculosis* O:1 and *L. monocytogenes* 2a and 4b were identified by culturing from the PCR-positive samples. *Brucella suis* biovar 2 was isolated from visceral organs. No African swine fever, classical swine fever or Aujeszky’s disease were detected in the wild boars. Our study shows that wild boars are important reservoirs of foodborne pathogens.

**Supplementary Information:**

The online version of this article (10.1007/s10393-020-01509-5) contains supplementary material, which is available to authorized users.

## Introduction

Wild boars (*Sus scrofa*), also known as wild pigs or feral pigs in the USA, have strongly expanded their range in northern Europe during the last decade (von Essen 2019). They are also very intensively hunted in Europe (Vajas et al. 2020) and the most important hunting species in the world (Massei et al. 2015). In Finland, the wild boar population has increased drastically during the past years with large regional differences in the population densities. Highest wild boar numbers have been reported in southeastern Finland near the Russian border, where approximately 25% of wild boars live (Natural Resources Institute Finland). A warming climate with milder winters and scarce snow is enabling the species to spread northwards and become more numerous (Markov et al. 2019). They will also continue to expand due to their high adaptability and high reproductive potential. Wild boars cause extensive agricultural damages and traffic accidents, but they are also a risk to human and animal health (Fredriksson-Ahomaa 2019).

Wild boars can be infected with several pathogens that are transmittable to other wildlife, domestic animals and humans (Fredriksson-Ahomaa 2019). Wild boars carry *Brucella suis, Salmonella*, *Yersinia enterocolitica* and *Y. pseudotuberculosis*, *Toxoplasma gondii*, *Trichinella* and hepatitis E virus (HEV) in Europe (Anheyer-Behmenburg et al. 2017; Kantala and Maunula. 2018; Bonardi et al. 2019; Laforet et al. 2019). Furthermore, *stx*-positive *Escherichia coli* (STEC) and *Listeria monocytogenes* have been detected in wild boar samples (Dias et al. 2019). These pathogens are all transmittable to humans through contaminated food. Wild boar meat is also recognized as an important source of *Trichinella* and *T. gondii*. A possible link of foodborne zoonoses between wild boars, domestic animals and humans has raised increasing interest among researchers (Bonardi et al. 2019). Wild boars may also act as reservoirs for many important livestock infectious diseases such as African swine fever (ASF), classical swine fever (CSF) and Aujeszky’s disease (AD) (Meier et al. 2015; Postel et al. 2018; Gallardo et al. 2019).

The purpose of this work was to study the prevalence of antibodies to *Salmonella*, pathogenic *Yersinia* spp., *T. gondii, Trichinella* and HEV, which are all important meat-borne pathogens associated with domestic and wild pig populations. Furthermore, *Salmonella*, *ail*-positive *Yersinia* spp., *Campylobacter* spp., STEC and *L. monocytogenes* were studied by PCR and culturing from the visceral organs. We additionally report the monitoring results for brucellosis, AFS, CSF and AD.

## Methods

### Samples

Finnish hunters collected samples (blood, spleen and kidneys) from 366 wild boars, which were intended for monitoring of brucellosis, ASF, CSF and AD, and sent them to the Finnish Food Safety Authority (the Finnish Food Authority from 1.1.2019 onward) between January and December 2016. Spleen and kidneys from each animal were shipped together in a plastic bag. We studied the prevalence of antibodies to the most important pork-borne zoonotic pathogens (*Salmonella, Yersinia, T. gondii, Trichinella* and HEV) from the serum samples. Furthermore, the presence of *Salmonella*, *Y. enterocolitica*, *Y. pseudotuberculosis*, *Campylobacter* spp., STEC and *L. monocytogenes* was studied from the visceral organ samples (spleen and kidneys) by PCR. Additionally, antibodies to *Brucella*, CSF virus (CSFV) and AD virus (ADV) were studied in wild boars. The presence of CSFV, ASF virus (ASFV) and ADV was studied using PCR.

### Serology

We studied antibodies to *Salmonella*, *Yersinia*, *T. gondii* and *Trichinella* in serum samples using commercial enzyme-linked immunosorbent assay (ELISA) Pigtype® test kits (Qiagen, Leipzig, Germany). According to the manufacturer, the sensitivity and specificity of the tests were 98.5% and 99.8% for *Salmonella*, 98.9% and 92.7% for *T. gondii* and 98.9% and 95.4% for *Trichinella*. For the *Yersinia* test, the sensitivity and specificity were both near 100% according to the manufacturer. Samples with an S/P ratio < 0.3 were considered negative. The PrioCHECK®HEV ab porcine ELISA test (91.0% sensitivity and 94.0% specificity) (Prionics AG, Zurich, Switzerland) was used for detecting HEV antibodies. The cutoff value was calculated according to the manufacturer’s instructions. Antibodies to *Brucella* were studied in serum samples using two tests: Rose Bengal (RB) rapid slide agglutination test (Pourquier® Rose Bengale Ag, IDEXX, France) and multi-species indirect ELISA test (sensitivity and specificity near 100%) (ID Screen® Brucellosis, IDVet, France). All samples were studied by RB and RB-positive samples also by ELISA. We studied the presence of antibodies to the CSFV using PrioCHECK®CSFV Antibody 2.0 ELISA assay (95.4% sensitivity and 100% specificity) (Prionics AG) and to the ADV using SVANOVIR® PRV gB-Ab ELISA assay (99.6% sensitivity and 99.3% specificity) (Svanova, Uppsala, Sweden).

### PCR Screening

We studied the presence of *Campylobacter*, *Salmonella*, *ail*-positive *Yersinia* spp., STEC and *L. monocytogenes* using real-time PCR based on SYBRGreen according to Sauvala et al. (2019). DNA was extracted from the organs after overnight enrichment [10 g samples in 90 ml of buffered peptone water (BPW, Labema, Helsinki, Finland)] at 37 °C for 10–20 h using ZR Fecal DNA MiniPred™ (Nordic BioSite Oy, Helsinki, Finland). We studied the presence of ASFV, CSFV and ADV using PCR according to Fernández-Pinero et al. (2013), Hoffmann et al. (2006) and Wernike et al. (2014), respectively.

### Isolation and Identification

PCR-positive *Campylobacter*, *Salmonella*, *Yersinia* and *Listeria* samples were isolated from the overnight enrichments using selective agar plates (Labema): CCDC and CHROMagar™Campylobacter for *Campylobacter*, XLD and CHROMagar™SalmonellaPLUS for *Salmonella*, CIN and CHROMagar™Yersinia enterocolitica for *Yersinia* and CHROMagar™Listeria for *Listeria*. Up to four typical colonies on selective agar plates were cultured on blood agar. All plates for *Salmonella, Campylobacter* and *Listeria* isolation were incubated at 37 °C and plates for *Yersinia* at 30 °C for 24–48 h. *Salmonella* and *Yersinia* colonies were identified with API 20E and *Listeria* colonies with API Listeria (BioMerieux, France). We studied the pathogenicity of the *Yersinia* isolates with PCR detecting the chromosomal *ail* gene and the plasmid-borne *vir*F gene. Serotyping was performed with commercial antisera (Denka Seikan, Japan). *Brucella* was isolated using Farrell’s selective agar plates (Oxoid, Basingstoke, UK) and Columbia blood agar plates containing bovine serum (Oxoid). The plates were incubated at 37 °C in an aerobic and microaerophilic atmosphere and observed for 10 d. Presumptive identification was based on Stamp staining and on the oxidase, catalase and urease tests. Further identification was performed with three PCR methods: (1) Genus identification was performed according to Bounaadja et al. (2009), and (2) species and (3) biovar were identified according to López-Goñi et al. (2011). *Brucella* isolation was performed in a biosafety level three (BSL-3) laboratory.

### Multi-locus Sequence Typing MLST

We purified the DNA of *Y. pseudotuberculosis* and *L. monocytogenes* isolates with PureLink Genomic DNA Mini Kit (Invitrogen, Carlsbad, USA). Whole-genome sequence was performed on the Illumina platform using NovaSeq6000 (Center for Genomics and Transcriptomics, Tuebingen, Germany) with paired end reads. Isolates were sequenced with 100 × coverage and 2 × 100 bp read length. Genome assembly was conducted using Patric web-based service (https://www.patricbrc.org/app/Assembly). The assembled contigs were analyzed with the web-based service of the Center for Genomic Epidemiology (https://cge.cbs.dtu.dk/services/MLST/): Sequence types (STs) of *Y. pseudotuberculosis* were determined with the MLST scheme for *Y. pseudotuberculosis,* and STs of *L. monocytogenes* were determined with the MLST scheme for *L. monocytogenes*.

### Statistical Analyses

Statistical analyses were carried out with the analytical software package SPSS® Statistics Version 25. Multiple logistic regression analysis was used to assess the association between potential risk factors (age and gender) and the presence of antibodies to foodborne pathogens. *P*-values below 0.05 were considered statistically significant.

## Results

A total of 181 wild boars, hunted in 12 out of 19 regions in Finland, were tested for antibodies to foodborne pathogens. Most (78%) of the 181 wild boars had been hunted in southeastern Finland, in region 7 (South Karelia) and region 16 (Kymenlaakso) (Figure S1). Both regions have the highest wild boar densities and a land border with Russia.

We obtained gender information for 177 out of 181 wild boars and age information for all 181 wild boars (Table 1). The animals were reported as young (below 1 year of age) or adults (over 1 year) based on tooth eruption (> 12 months of age: 2. molar has erupted). Adult males (*n* = 49) were slightly more common than young males (*n* = 44), and young females (*n* = 45) were slightly more common than adult females (*n* = 39).

**Table 1:** Geographical Locations (Regions) of the 181 Hunted Wild Boars.

Region	Number of wild boars		Gender			Age	
			Male	Female	NR^b^	Adult	Young
2	4	(5)^a^	2	1	1	1	3
3	1	(2)	1	0	0	1	0
4	9	(15)	3	6	0	6	3
5	2	(8)	1	1	0	2	0
6	1	(6)	1	0	0	1	0
7	83	(126)	41	40	2	35	48
8	3	(18)	1	2	0	1	2
12	3	(6)	1	2	0	1	2
13	0	(1)					
14	2	(14)	2	0	0	1	1
15	1	(2)	0	1	0	1	0
16	58	(125)	31	26	1	31	27
17	14	(35)	9	5	0	7	7
18	0	(3)					
All	181	(366)	93	84	4	88	93

^a^Number of wild boars obtained for the monitoring.

^b^Not reported.

The number of wild boars studied per month varied between 0 and 50 animals (Figure S2). No samples were obtained from April to May, which is the farrowing time when fewer wild boars are hunted. The highest number (*n* = 50) was obtained in November.

Antibodies to *Salmonella* and *Yersinia* were found in 38% (69/181) and 56% (102/181) of the wild boars, respectively (Table 2). HEV antibodies were found in 18% (32/181) wild boars. *T. gondii* antibodies were detected in 9% (17/181) and *Trichinella* antibodies in 1% (2/181) of the wild boars. Antibodies to pathogens, especially *Salmonella, Yersinia* and HEV, were frequently found in wild boars hunted in regions 7 and 16 in southeastern Finland.

**Table 2:** Seroprevalence of Foodborne Zoonoses in Wild Boars Hunted in 12 Regions in Finland.

Region	Number of wild boars	Number of wild boars with antibodies to				
		*Salmonella*	*Yersinia*	*Toxoplasma*	*Trichinella*	HEV
2	4	1	3	0	0	0
3	1	0	1	1	0	0
4	9	3	7	2	0	3
5	2	0	2	0	0	0
6	1	1	0	0	0	0
7	83	33	41	4	1	14
8	3	0	0	0	0	0
12	3	1	3	0	0	1
14	2	1	1	1	0	0
15	1	0	1	0	0	1
16	58	25	34	6	1	10
17	14	4	9	3	0	3
Total	181	69 (38%)	102 (56%)	17 (9%)	2 (1%)	32 (18%)
95% confidence intervals		31%-46%	49%-64%	6%-15%	1%-4%	12%-24%

The seroprevalence of *Salmonella, Yersinia, T. gondii*, *Trichinella* and HEV infections did not differ significantly (*P* > 0.05) between male and female wild boars (Table 3). However, males were 2.3 times more likely to carry antibodies to any of these pathogens than females (*P*= 0.017). Age significantly influenced the presence of *Yersinia* (*P*= 0.021) and *T. gondii* (*P*= 0.011) antibodies; adult wild boars were 2.0 times more likely to carry *Yersinia* antibodies and 5.3 times more likely to carry *T. gondii* antibodies than young wild boars. *Trichinella* antibodies were detected in two wild boars: Both were adult females.

**Table 3:** Prevalence of Antibodies to Foodborne Zoonotic Pathogens in Young and Adult/Female and Male Wild Boars.

Antibodies to	Variables		Seropositivity N (%)	Odds ratio (CI95%)^a^	*P*-value^b^
*Salmonella*	Age	Young	34 (39%)		> 0.05
		Adult	34 (39%)		
	Gender	Female	28 (33%)		> 0.05
		Male	40 (43%)		
*Yersinia*	Age	Young	43 (48%)	2.04 (1.11–3.75)	0.021
		Adult	58 (66%)		
	Gender	Female	45 (54%)		> 0.05
		Male	65 (60%)		
*Toxoplasma*	Age	Young	3 (3%)	5.30 (1.46–19.21)	0.011
		Adult	14 (16%)		
	Gender	Female	6 (7%)		> 0.05
		Male	11 (12%)		
*Trichinella*	Age	Young	0		> 0.05
		Adult	2 (2%)		
	Gender	Female	2 (2%)		> 0.05
		Male	0		
HEV	Age	Young	15 (16%)		> 0.05
		Adult	17 (19%)		
	Gender	Female	15 (18%)		> 0.05
		Male	17 (18%)		
Any pathogen	Age	Young	63 (71%)		> 0.05
		Adult	66 (75%)		
	Gender	Female	54 (64%)	2.29 (1.16–4.54)	0.017
		Male	75 (81%)		

^a^Multiple logistic regression analysis.

^b^*P*-value < 0.05 is considered significant.

*L. monocytogenes* (48%, 63/130) and STEC (33%, 43/130) determinants were the most common findings in wild boars with PCR (Table 4). *Yersinia* carrying the *ail* gene was detected in 17% (22/130) of the animals, while *Salmonella* and *Campylobacter* determinants were found in 5% (6/130) of the individuals by PCR.

**Table 4:** Detection Rate of Foodborne Pathogens in Wild Boars by PCR.

Region	Number of wild boars	Wild boar positive for genes				
		*rrn*	*ttr*	*ail*	*mpl*	*stx*
2	3	0	0	0	3	3
3	1	0	0	0	0	0
4	6	0	1	1	3	3
5	1	0	0	0	0	0
7	66	6	2	12	26	23
12	3	0	0	0	2	2
14	2	0	0	0	1	0
15	1	0	0	0	0	0
16	36	0	2	7	21	9
17	11	0	1	2	7	3
Total	130	6 (5%)	6 (5%)	22 (17%)	63 (48%)	43 (33%)

*rrn* = *Campylobacter*, *ttr* = *Salmonella*, *ail* = *Yersinia*, *mpl* = *L. monocytogenes*, *stx* = STEC

*L. monocytogenes* and *Y. pseudotuberculosis* could be isolated from the visceral organs (spleen and kidneys) (Table 5). Fifty-two *L. monocytogenes* isolates originated from 40 (30%) wild boars. Nearly all *L. monocytogenes* isolates belonged to serotype 2a, but two isolates from two wild boars were identified as serotype 4b. Several STs were obtained from *L. monocytogenes* isolates. *Y. pseudotuberculosis* was isolated from two wild boars, both of which were young females from region 7 (South Karelia). The isolates belonged to serotype O:1 and ST42.

**Table 5:** *Listeria monocytogenes* and *Yersinia pseudotuberculosis* Isolates from Wild Boars.

Region	*Listeria monocytogenes*				*Yersinia pseudotuberculosis*			
	Isolates (Animals)		Serotypes	Sequence types (STs)	Isolates (Animals)		Serotype	Sequence type (ST)
4	2	(1)	2a		0			
7	29	(21)	2a, 4b	1, **18**^a^, 20, 21, 91, 399, **451**	3	(2)	O:1	42
12	1	(1)	2a		0			
14	3	(3)	2a	**451**	0			
16	12	(10)	2a	8, **37**, **451**, 573	0			
17	5	(4)	2a	**7**	0			
Total	52	(40)			3	(2)		

^a^Bold STs have been found on moose and deer carcasses in Finland (Sauvala et al. 2019).

Antibodies to *Brucella* were detected in 9% (8/87) of wild boars. Visceral organs were available from 5 out of 8 seropositive animals of which *B. suis* biovar 2 was isolated from 4 wild boars. All positive animals were hunted in South Karelia (region 7), which is located in southeastern Finland and has a long land border with Russia. No ASFV was detected in 366 wild boars by PCR. Furthermore, no CSFV was detected in 230 and 366 wild boars and ADV in 234 and 362 wild boars by serology and PCR, respectively.

## Discussion

A high seroprevalence (38%) of *Salmonella* infection was detected in wild boars hunted in Finland in 2016. *Salmonella* prevalence reached 40% and 43%, in southeastern regions 7 and 16 with Russian land border, respectively. Only a few serological studies have recently been conducted on *Salmonella* in wild boars (Fredriksson-Ahomaa 2019). In Spain, a seroprevalence of 11% was reported in wild boars quite recently (Cano-Manuel et al. 2014). Interestingly, *Salmonella* was detected in 27% of wild boars in Sweden using PCR, mostly in the tonsils (Sannö et al. 2018). There are no data available about *Salmonella* from wild boars in Finland. Wild boars frequently carry *Salmonella* in their tonsils, but *Salmonella* also colonizes the lymph nodes and is excreted in the feces (Sannö et al. 2018; Bonardi et al. 2019; Gil Molino et al. 2019). In Belgium, *Salmonella* was recently detected in one third of wild boar meat samples (Peruzy et al. 2019). *Salmonella* and other bacteria can easily be transmitted from the tonsils and feces to the carcass and visceral organs during evisceration, especially due to poor hunting hygiene out in the field or due to gut hit (Sauvala et al. 2019). In our study, *Salmonella* determinant (*ttr*) was detected in 5% of the visceral organs using PCR, but no *Salmonella* was found by culturing indicating a low contamination level of the organs. The organs have also been stored at − 20 °C for a prolonged time before culturing, which could have influenced the viability of the cells. Unfortunately, we did not test feces or tonsil samples, which are the matrices of choice to estimate *Salmonella* prevalence in wild boars (Sannö et al. 2014). However, slaughter waste should not be left out in the forest because it may pose the risk of *Salmonella* spreading to other wild and domestic animals. Finland, Sweden and Norway have very low *Salmonella* isolation rates in domestic pigs (< 1%) compared to other European countries (EFSA and ECDC 2018). Biosecurity in pig farms is crucial for reducing the risk of introducing *Salmonella* from wildlife into the pork production chain.

The seroprevalence of *Yersinia* infection in wild boars was high (56%) in our study. A high seroprevalence has also been reported in the Czech Republic (66%), Latvia (69%) and Spain (52%) showing that wild boars are frequently infected with *Yersinia* (Arrausi-Subiza et al. 2016; Lorencova et al. 2016; Grantina-Ievina et al. 2018). In our study, antibodies to *Yersinia* were significantly more frequently detected in adults than in young wild boars, which is in accordance with a study from Spain (Arrausi-Subiza et al. 2016). *Y. enterocolitica* and *Y. pseudotuberculosis* have been detected particularly in the tonsils, constituting a contamination risk for carcasses during slaughter (Wacheck et al. 2010; Sannö et al. 2014, 2018; Reinhardt et al. 2018). In our study, 17% of the visceral organs were contaminated with *ail*-positive *Yersinia*; however, only *Y. pseudotuberculosis* serotype O:1 was isolated from the samples. Serotype O:1 belonging to the sequence type ST42 is a common type reported in wild boars (Fredriksson-Ahomaa et al. 2011; Reinhardt et al. 2018). This type has also been identified in humans in Europe (http://enterobase.warwick.ac.uk/species/index/yersinia). Seroprevalence of yersiniosis is also high in fattening pigs in Europe including Finland (Felin et al. 2019). However, the etiology seems to differ between domestic pigs and wild boars. The infection agent in domestic pigs is typically *Y. enterocolitica* of bioserotype 4/O:3, and *Y. pseudotuberculosis* is a rare finding. Wild boars seem to be infected with *Y. pseudotuberculosis* and *Y. enterocolitica* belonging to different genotypes than domestic pigs (Fredriksson-Ahomaa et al. 2011). Domestic pigs are usually infected during the fattening period, and their seroprevalence decreases with age, being low in sows. In wild boars, seroprevalence was significantly higher in adult than in young animals indicating infection or reinfection at an older age.

HEV is the most important meat-borne zoonotic virus. Wild boars are an important reservoir of HEV in Europe (Kantala and Maunula 2018; Fredriksson-Ahomaa 2019). Antibodies to HEV were detected in 18% of wild boars in our study. A seroprevalence of over 50% has quite recently been reported in Germany, Italy, Lithuania and Spain (Mazzei et al. 2015; Kukielka et al. 2016; Spancerniene et al. 2016; Anheyer-Behmenburg et al. 2017). Foodborne HEV transmission has been associated with the liver, meat and sausages of wild boar (Fredriksson-Ahomaa 2019). A high HEV load (> 10^5^ genomic copies/g) has recently been reported in wild boar liver in Italy (Di Pasquale et al. 2019). Genetically highly related HEV strains have been reported among humans, domestic pigs and wild boars in European countries indicating interspecies transmission of HEV (Jemeršic et al. 2019; Wang et al. 2019). A high prevalence of hepatitis E among wild boars and the similarity of HEV strains between wild boars and humans indicate that zoonotic transmission from wild boars may be more common than previously expected (Wang et al. 2019).

We observed a 9% seroprevalence of *T. gondii* infection in wild boar. A clearly higher seroprevalence (50%) was reported in Sweden (Wallander et al. 2015). One reason for this may be the higher wild boar density and more frequent contact with cat feces in Sweden compared to Finland. *T. gondii* antibodies were detected in 33% of farmed wild boars in Finland (Jokelainen et al. 2012) but only in 1% of fattening pigs bred under a controlled housing system (Felin et al. 2019). A *T. gondii* seroprevalence of 28% in fenced wild boars in Denmark was recently reported (Laforet et al. 2019). A high seroprevalence of ca. 40% was reported in wild boars in the Czech Republic, Italy and Slovakia (Racka et al. 2015; Reiterová et al. 2016; Gazzonis et al. 2018). In our study, *T. gondii* antibodies were significantly more frequently detected in adults than in young wild boars, which is in accordance with earlier studies (Fredriksson-Ahomaa 2019). A higher prevalence in older than in young animals is most probably due to their greater exposure to the parasite. Recently, *T. gondii* was frequently reported in wild boar tissue samples (brain: 31%, heart: 28% and masseter muscle: 24%) in southern Italy (Santoro et al. 2019). Consumption of undercooked wild boar meat is expected to be a risk for contracting human toxoplasmosis. However, since *T. gondii* is sensitive to freezing (Jones and Dubey et al. 2012), only frozen wild boar meat should be used for consumption, if not thoroughly heat treated. Jones and Dubey (2012) recommend cooking wild boar meet to 71 °C or higher with a 3-min rest to avoid toxoplasmosis.

Antibodies to *Trichinella* were detected in only 1% of the wild boars in our study. An exceptionally high seroprevalence of 42% has been reported in Estonia (Kärssin et al. 2016). Before the AFS epidemic in 2014, wild boar density was very high in Estonia, allowing close interaction between wild boars and other wild animals. Wild boar meat has recently been responsible for several human *Trichinella* outbreaks in Europe (Faber et al. 2015; Fichi et al. 2015; Van De et al. 2015; Messiaen et al. 2016; Heaton et al. 2018). Wild boar carcasses should be appropriately tested to prevent human exposure to *Trichinella* (Noeckler et al. 2019). *Trichinella* testing is required for all wild boars entering the community trade in Europe according to the legislation (Regulations (EC) No 853/2004). Freezing only is not recommended because *T. native* and *T. britovi* are relatively freeze resistant (Noeckler et al. 2019). These two species are the most common ones identified in wildlife in Finland (Oksanen et al. 2018). Cooking at core temperature between 63 °C and 71 °C for 3 min inactivates *Trichinella* larvae in meat (Noeckler et al. 2019).

We also studied the presence of *Campylobacter*, STEC and *L. monocytogenes* determinants in the visceral organs of wild boars using PCR and culturing. We used PCR to screen positive samples before culturing because it is a more rapid, sensitive and specific method compared to culturing. However, PCR detects also dead cells, which can cause false positive results. The PCR prevalence of *Campylobacter* was 5%, and no isolates were found by culturing. Furthermore, *Campylobacter* will usually not survive on a dry carcass surface and is heat sensitive (Nesbakken et al. 2008, Silva et al. 2011). This indicates that wild boar meat is a relatively low risk of *Campylobacter* infection. A high PCR prevalence of STEC and *L. monocytogenes* determinants was detected in the visceral organs. Bacterial contamination of game carcasses and organs occurs easily during evisceration in the field (Sauvala et al. 2019). STEC was studied only with a PCR method targeting the *stx*1 and *stx*2 genes and not by culturing; further investigations are thus needed to confirm the public health relevance. The isolation rate of *L. monocytogenes* was surprisingly high. Serotypes 2a and 4b identified in wild boars have also been found on moose and deer carcasses in Finland (Sauvala et al. 2019). Several sequence types were obtained, most of which have been identified in humans in Europe (https://bigsdb.pasteur.fr/listeria/). Sequence types ST7, ST8, ST18, ST37 and ST451 of serotype 2a and ST1 of serotype 4b found in wild boars in Finland have also been found in humans with listeriosis (Finnish Institute for Health and Welfare). The link between wild boar/wildlife and human infections needs further studies. However, *L. monocytogenes* is tolerant to cold. It has an ability to grow at temperatures around 0 °C, and therefore, storing wild boar meat in a refrigerator may present a public health risk (Hingston et al. 2017).

Antibodies to *Brucella* were found in 9% of the wild boars in Finland in 2016. Antibodies to *Brucella* have been detected in wild boars in several European countries: A very high seroprevalence was reported in Spain, while no antibodies to *Brucella* were reported in Sweden (Fredriksson-Ahomaa 2019). Several European countries have achieved *Brucella*-free status in livestock. This includes Finland, where *Brucella* has never been reported in domestic pigs. *B. suis* biovar 2, which was found in wild boars in Finland for the first time in 2015, was also found in 2016. *B. suis* biovar 2 is the most common type responsible for brucellosis in wild boars and hares in Europe (De Massis et al. 2019; Muñoz et al. 2019). Brucellosis may cause major economic losses in the pig industry, especially in pigs with outdoor access. Brucellosis due to *B. suis* biovar 2 has been reported in hunters, most probably due to direct contact with organs and tissues of infected wild boars (Mailles et al. 2017).

Several animal diseases, such as ASF, CSF and AD, are monitored in domestic pigs and wild boars in Finland. Wild boars hunted in Finland in 2016 were negative for these contagious diseases. However, wild boars are important reservoir of ASFV, CSFV and ADV in several European countries and may also transmit these pathogens to domestic pigs causing tremendous economic losses in the pig production chain (Meier et al. 2015; Postel et al. 2018; Gallardo et al. 2019). ASF has so far never been detected Finnish domestic pigs or wild boars, and CFS has not been detected since 2017. ASF has been detected in regions close to Finland, and the risk of its spread to Finland has increased (Finnish Food Authority). Wild boars have been monitored for ASF since 2010, and all wild boars found dead should be reported to local municipal veterinarians soon as possible. AD has never been diagnosed in Finnish domestic pigs. However, AD was reported for the first time in a wild boar in September 2019. It was hunted in the North Karelia (region 4), close to the Russian border. Effective biosecurity measures at the farm level and a low wild boar density are very important in preventing the spread of contagious diseases to pig farms and resulting in huge economic losses in the pig production chain.

## Conclusion

Wild boars are important reservoirs for foodborne zoonoses. Our study demonstrated a high seroprevalence of *Salmonella* and *Yersinia* infections, but we also detected antibodies to *Brucella*, *T. gondii, Trichinella* and HEV in hunted wild boars. Wild boars may transmit these foodborne pathogens to other animals and hamper the control of these pathogens in the food-producing chain. *L. monocytogenes* was frequently detected in the visceral organs stressing the importance of proper hygiene when handling wild boar organs. Monitoring of infectious diseases in wild boars may help to implement locally customized public and animal health control strategies to avoid financial losses in food production chain.

## Electronic supplementary material

Below is the link to the electronic supplementary material.

Supplementary material 1 (DOCX 571 kb)
