# Correction to: Foodborne Zoonoses Common in Hunted Wild Boars

**DOI:** 10.1007/s10393-022-01585-9

**Published:** 2022-03-18

**Authors:** Maria Fredriksson-Ahomaa, Laura London, Teresa Skrzypczak, Tuija Kantala, Ilona Laamanen, Mia Biström, Leena Maunula, Tuija Gadd

**Affiliations:** 1 Department of Food Hygiene and Environmental Health, Faculty of Veterinary Medicine, University of Helsinki, P.O.Box 66, 00014 Helsinki, Finland; 2 Virology Unit, Finnish Food Authority, Helsinki, Finland; 3 Veterinary Bacteriology and Pathology Unit, Finnish Food Authority, Helsinki, Finland

## Correction to: EcoHealth 17, 512–522, 2020 10.1007/s10393-020-01509-5

In the sentence beginning ‘Several animal diseases, such as ASF, CSF and AD, are monitored in domestic pigs and wild boars in Finland’ in this article, the year ‘2017’ should have read ‘1917'.

