# Supplementary material for: Foodborne Zoonoses Common in Hunted Wild Boars
Source: Ecohealth. 2020 Dec 16;17(4):512–22. doi: 10.1007/s10393-020-01509-5 (PMC8192372; doi:10.1007/s10393-020-01509-5)
Supplement: Supplementary file 1 — Supplementary material 1 (DOCX 571 kb) [file 10393_2020_1509_MOESM1_ESM.docx]

Supplementary data


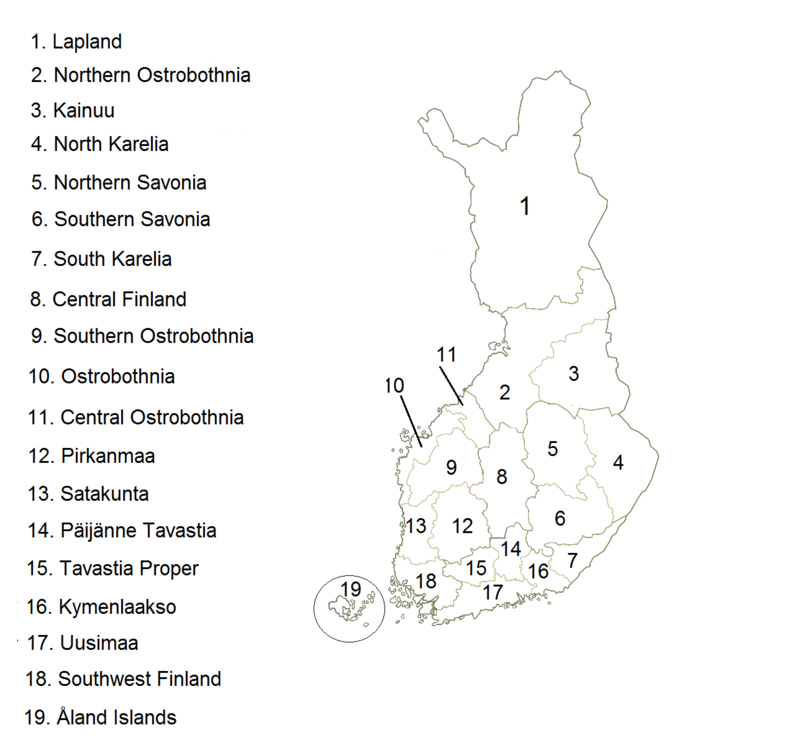


1. Lapland
2. North Ostrobothnia
3. Kainuu
4. North Karelia
5. North Savonia
6. South Savonia
7. South Karelia
8. Central Finland
9. South Ostrobothnia
10. Ostrobothnia
11. Central Ostobothnia
12. Pirkanmaa
13. Satakunta
14. Päijänne Tavastia
15. Tavastia Proper
16. Kymenlaakso
17. Uusimaa
18. Southwest Finland
19. Åland Island

By Paasikivi - Own work, CC BY-SA 4.0, https://commons.wikimedia.org/w/index.php?curid=56079832

Figure S1. Locations of the regions in Finland.


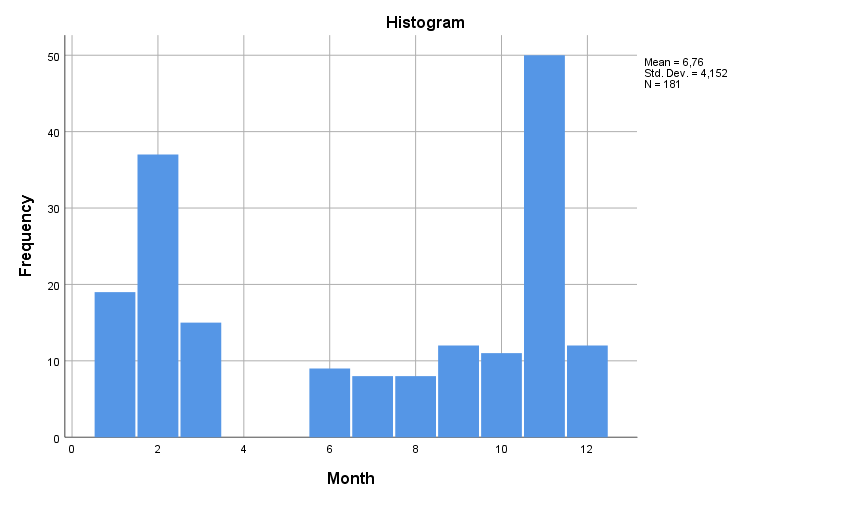


Jan Feb Mar Jun Jul Aug Sep Oct Nov Dec

Figure S2. Number of hunted wild boars per month in 2016.
